# Insulin and Incretin Receptor Agonists Reciprocally Alter Their Blood–Brain Barrier Permeabilities

**DOI:** 10.3390/ijms27104611

**Published:** 2026-05-21

**Authors:** Angeline Fry, Alexis Rose, Riley Weaver, Kim Hansen, James E. Blevins, William A. Banks, Elizabeth M. Rhea

**Affiliations:** 1 Geriatric Research Education and Clinical Center, Veterans Affairs Puget Sound Health Care System, Seattle, WA 98108, USA; 2 Office of Research and Development, Veterans Affairs Puget Sound Health Care System, Seattle, WA 98108, USA; 3 Division of Metabolism, Endocrinology and Nutrition, Department of Medicine, University of Washington, Seattle, WA 98195, USA; 4 Division of Gerontology, Department of Medicine, University of Washington, Seattle, WA 98195, USA

**Keywords:** incretin receptor agonists, GLP-1 receptor agonists, insulin, blood–brain barrier

## Abstract

Incretin receptor agonists (IRAs) such as GLP-1-based therapies improve metabolic and cognitive outcomes and enhance brain insulin signaling. One way that IRAs could have these actions is by affecting the blood–brain barrier (BBB); however, IRA-BBB interactions are poorly studied. Here, we examined the ability of insulin and IRAs to affect each other’s transport across the BBB in lean mice. We found that intracerebroventricular (ICV) administration of the insulin receptor antagonist S961 did not affect the blood-to-brain transport of the bioactive fragment of the IRA, ^125^I-dulaglutide (BAF). In contrast, ^125^I-dulaglutide (BAF) co-administered with intravenous (IV) insulin significantly enhanced ^125^I-dulaglutide (BAF) BBB transport into whole brain, olfactory bulb, parietal cortex, and pons, demonstrating insulin-dependent modulation of IRA BBB transport. Regional transport rates for ^125^I-dulaglutide (BAF) across the brain varied by ~2.5-fold, with the fastest transport into the olfactory bulb, frontal cortex, cerebellum, and pons. Co-administration of IV dulaglutide (BAF) did not alter ^125^I-insulin BBB transport rates (*K_i_*) but did reduce reversible insulin binding (*V_i_*) at the BBB by >50%, suggesting rapid effects on BBB insulin receptors. To explore the effects of chronic IRA administration, lean mice were treated with semaglutide for two weeks. Body weight and food intake were unchanged, but female mice showed reduced fasting levels of serum insulin and GLP-1 and decreased insulin transport into whole brain, while male mice showed a reduction in insulin binding at the BBB. Chronic semaglutide also reduced ^125^I-insulin BBB transport in female mice when studied with in situ perfusion, a procedure that removes the immediate influence of serum factors. Together, these findings demonstrate reciprocal and female-selective interactions between IRAs and insulin at the BBB. Acute insulin enhances the BBB transport of an IRA in female mice, whereas chronic IRA exposure selectively impairs insulin BBB transport in females, highlighting the BBB as a dynamic and hormone-sensitive interface with implications for long-term treatment in mouse models and potential for translation impact in humans.

## 1. Introduction

A frequent feature of Alzheimer’s disease (AD) is dysregulated brain insulin signaling, which through reduced neuronal function and survival contributes to cognitive impairment. Antidiabetic incretin receptor agonist (IRA) therapies not only reduce systemic insulin resistance in obesity and type 2 diabetes but also restore brain insulin signaling and reverse associated neurodegenerative characteristics of AD in animal models. However, the mechanisms driving IRA-induced enhancement of brain insulin signaling remain poorly understood.

Originally developed for the treatment of type 2 diabetes, IRAs have garnered increasing interest for their potential neuroprotective effects in aging and neurodegenerative diseases. Epidemiological and clinical studies indicate that IRA treatment is associated with a reduced risk of cognitive decline and dementia, including AD. Large population-based analyses have shown that individuals with type 2 diabetes treated with IRAs exhibit a lower incidence of all-cause dementia and AD compared with those receiving other glucose-lowering therapies such as SGLT2 and DPP4 inhibitors [1,2,3]. Additional clinical trials have been completed in individuals with AD without diabetes. A multicenter, placebo-controlled clinical trial, ‘Evaluating liraglutide in Alzheimer’s disease’ (ELAD), identified that despite no significant differences in the cerebral glucose metabolic rate (primary outcome), cognition was improved as assessed by the AD Assessment Scale-Executive domain (ADAS-Exec, secondary outcome) [4]. In the industry-sponsored EVOKE trial investigating oral semaglutide in individuals with AD, there was no significant change in cognitive measures following 104 weeks of treatment, as assessed via the clinical dementia rating scale, and the trial was terminated early [5]. Unanalyzed data in a subset of participants at week 156 show a separation between the treatment groups, with the semaglutide group exhibiting less worsening in cognitive tests. Whether the beneficial effects occur if treated during the prodromal stage of AD remains to be tested, and the most responsive patient population to target remains to be identified. These findings are particularly compelling given the strong epidemiological links between insulin resistance, type 2 diabetes, and increased AD risk, positioning IRAs as candidates for disease-modifying interventions at the metabolic–neurodegenerative interface.

Preclinical studies provide mechanistic support for the cognitive benefits of IRA therapy. GLP-1 receptors are expressed in neurons, astrocytes, and microglia, and their activation improves synaptic plasticity, reduces neuroinflammation, and enhances insulin signaling within the brain [6,7,8,9,10,11]. In transgenic mouse models of AD, IRAs such as exenatide, liraglutide, and semaglutide reduce amyloid-β and tau pathology, preserve hippocampal synapses, and improve learning and memory [12,13,14]. Collectively, these clinical and experimental findings support the hypothesis that IRAs confer neuroprotective effects and may modify dementia risk, underscoring the importance of understanding how these therapies access and influence the brain.

Some IRAs readily cross the blood–brain barrier (BBB), whereas others cross poorly at best [15,16,17]. We previously identified that the transport rate for the bioactive fragment (BAF) of dulaglutide (1.095 µL/g-min) is over 2000 times greater than that for semaglutide (0.0005 µL/g-min) [17]. Semaglutide is designed to bind albumin, decreasing the clearance rate from blood. Albumin binding, however, reduces the amount of free drug available for transport and so contributes to the slower BBB transport rate, with transport rates for semaglutide being similar to that for albumin [17]. However, what is less well known is whether IRAs can affect the function of the BBB by altering peptide transport for other key hormones involved in insulin signaling, particularly insulin itself. Such a BBB interaction would occur on the luminal side of the brain endothelial cell and so would not require IRAs to cross the BBB to reach a site of action.

To better understand how IRAs mechanistically attenuate dysregulated brain insulin signaling and ameliorate AD disease processes, this study examined dulaglutide (BAF) and semaglutide BBB transport regulation and whether these IRAs can impact insulin BBB transport after acute and chronic administration in lean adult mice. We also examined the effect of insulin signaling on dulaglutide (BAF) penetration of the BBB. We hypothesized that treatment with these IRAs would enhance insulin BBB interactions (transport and binding), elucidating a mechanism for enhanced brain insulin signaling.

## 2. Results

### 2.1. Effect of Brain Insulin Receptor Inhibition on Dulaglutide (BAF) BBB Transport

**Figure 1:**
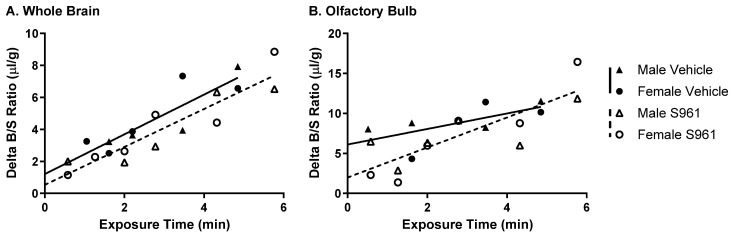
Effect of acute brain insulin receptor inhibition on ^125^I-dulaglutide (BAF) BBB pharmacokinetics. Delta ^125^I-dulaglutide (BAF) pharmacokinetics were measured for (**A**) whole brain (*n* = 9–11) and (**B**) olfactory bulb (*n* = 7–12) 10 min after a 1 µL ICV injection of vehicle or S961 (1 µg). Males (triangles) and females (circles) were combined for the linear regression (Vehicle—solid line, S961—dotted line).

We previously determined that acute inhibition of the insulin receptor in the brain with the insulin antagonist S961 slowed insulin BBB transport in female mice [18], highlighting a critical sex-selective feedback mechanism from the brain to the BBB. Since IRAs are known to enhance brain insulin signaling, we wanted to examine whether brain insulin signaling could also regulate IRA BBB transport. Using the paradigm that showed brain insulin receptor blockade slowed insulin transport across the BBB [18], we injected S961 (1 µg), a selective inhibitor of the insulin receptor, directly into the lateral ventricle 10 min before assessing ^125^I-dulaglutide (BAF) BBB transport (Figure 1, Table 1). There were no changes in ^125^I-dulaglutide (BAF) BBB transport into whole brain or the olfactory bulb following inhibition of the brain insulin receptor in male and female mice combined. This indicates that the same brain insulin receptor signaling feedback process regulating insulin BBB transport is not involved in regulating IRA BBB transport.

### 2.2. Effect of Insulin on Dulaglutide (BAF) BBB Transport

**Figure 2:**
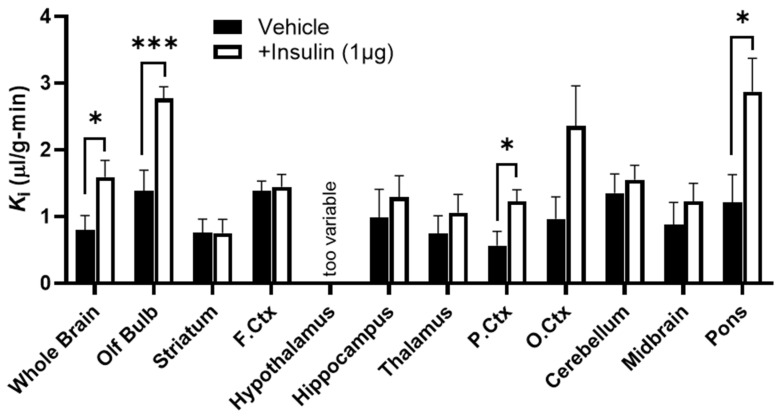
Effect of IV insulin co-administration on ^125^I-dulaglutide (BAF) BBB pharmacokinetics. Delta ^125^I-dulaglutide (BAF) pharmacokinetics were measured in male and female mice combined for multiple brain regions in the presence or absence of co-administered IV insulin (1 µg). Linear regressions for each region within each group were calculated, and the resulting slope (*K*_i_) is presented. Full linear regression data and sample size are listed in Table 2. “Too variable” indicates that the linear regression data was too variable and not significant. * *p* < 0.05, *** *p* < 0.001.

We then determined whether insulin signaling would affect IRA transport across the BBB. We found that insulin (1 µg) co-administration in the IV injection with ^125^I-dulaglutide (BAF) increased the BBB transport of ^125^I-dulaglutide (BAF) in some brain regions, including the whole brain, olfactory bulb, parietal cortex, and pons in male and female mice combined (Figure 2, Table 2). These data indicate that acute insulin administration can affect IRA BBB transport rates.

In this same study, we also saw regional variation in IRA BBB transport rates. ^125^I-dulaglutide (BAF) BBB transport was fastest for the olfactory bulb, frontal cortex, cerebellum, and pons (Table 2). Transport was slowest in the parietal cortex and thalamus.

### 2.3. Effect of Dulaglutide (BAF) on Insulin BBB Transport

**Figure 3:**
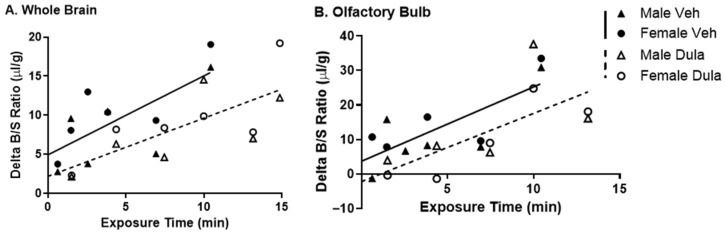
Effect of IV dulaglutide (BAF) co-administration on ^125^I-insulin BBB pharmacokinetics. Delta ^125^I-insulin pharmacokinetics were measured for (**A**) whole brain (*n* = 12) and (**B**) olfactory bulb (*n* = 11–12) in the presence or absence of co-administered IV dulaglutide (BAF) (1 µg). Males (triangles) and females (circles) were combined for the linear regression (Vehicle—solid line, Dulaglutide (BAF)—dotted line). Veh: vehicle, Dula: dulaglutide (BAF).

Because we identified that dulaglutide (BAF) BBB transport is regulated by acute increases in insulin levels, we wanted to examine whether dulaglutide (BAF) could affect ^125^I-insulin BBB transport. Dulaglutide (BAF) (1 µg) co-administered with the IV injection of ^125^I-insulin did not significantly alter ^125^I-insulin BBB transport rates (*K_i_*) into the whole brain or olfactory bulb but did significantly reduce the binding (*V_i_*) of insulin to the BBB by over 50% in males and females combined (Figure 3, Table 3). These data indicate that acute IRA administration does not affect the transport rate for insulin across the BBB but may affect the reversible binding at the BBB.

### 2.4. Effect of Chronic Semaglutide on Insulin BBB Transport

**Figure 4:**
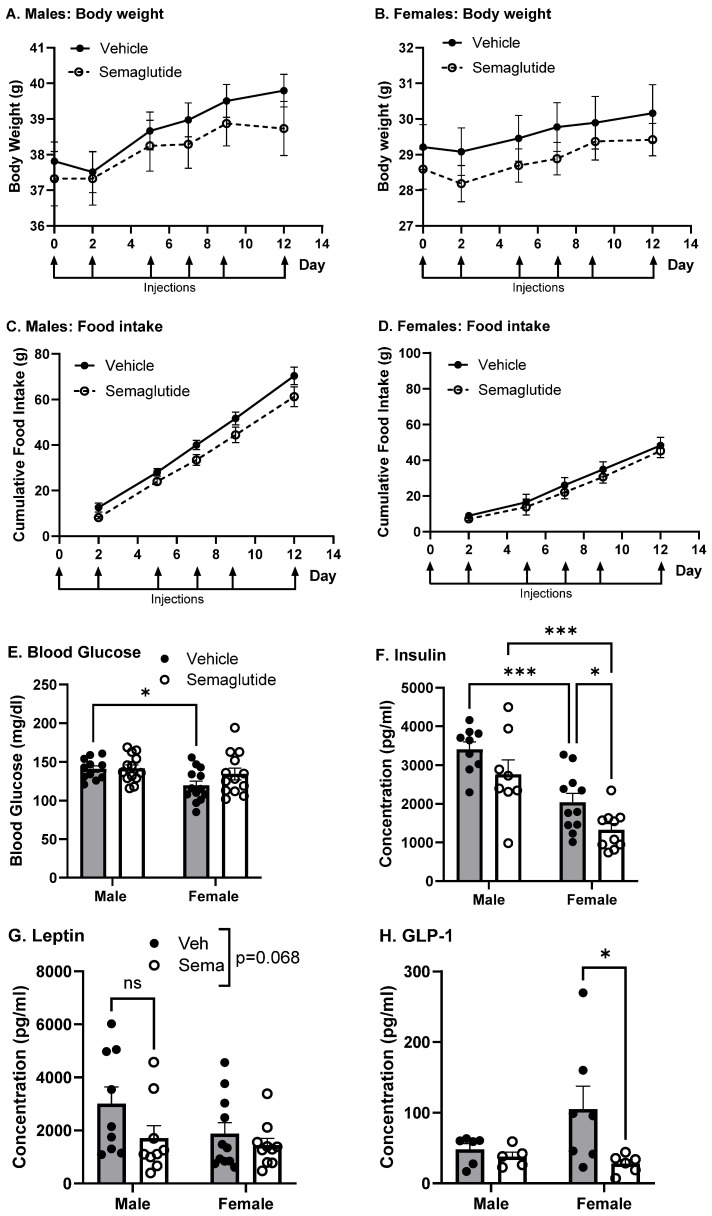
Metabolic effects of chronic semaglutide treatment. Male and female lean mice were treated for two weeks with s.c. semaglutide (dose escalation to 10 nmol/kg). (**A**,**B**) Body weights (*n* = 19 male, *n* = 20 female) and (**C**,**D**) food intake (*n* = 5 cages male, *n* = 3 cages female) were measured each injection day. Fasting (**E**) blood glucose (*n* = 11 male Veh, *n* = 13 female Veh, *n* = 13 male Sema, *n* = 13 female Sema), (**F**) insulin (*n* = 9 male Veh, *n* = 11 female Veh, *n* = 8 male Sema, *n* = 10 female Sema), (**G**) leptin (*n* = 9 male Veh, *n* = 11 female Veh, *n* = 9 male Sema, *n* = 10 female Sema), and (**H**) GLP-1 (*n* = 6 male Veh, *n* = 7 female Veh, *n* = 5 male Sema, *n* = 6 female Sema) were measured in a subset of mice from the three combined cohorts at the end of the treatment period (*n* = 11 male Veh, *n* = 13 male Sema, *n* = 13 female Veh, *n* = 13 female Sema). Two-way ANOVA with Fisher’s uncorrected LSD post hoc test: * *p* < 0.05, *** *p* < 0.001, ns = not significant, *p* < 0.10. Grey bars = vehicle treated, white bars = semaglutide treated. Veh: vehicle, Sema: semaglutide.

Since we observed significant effects of acute IRA administration on insulin BBB pharmacokinetics, we wanted to determine whether chronic IRA treatment elicited significant effects. Chronic IRA treatment is more translationally relevant, as individuals prescribed IRAs take them chronically for beneficial effects on type 2 diabetes and weight loss. To understand the functional effects on the BBB following chronic IRA treatment, we treated adult, lean mice for two weeks approximately every other day with s.c. semaglutide or vehicle. To avoid gastrointestinal side effects related to semaglutide treatment, we used a dose-escalation paradigm, beginning with 1 nmol/kg for the first injection, 3 nmol/kg for the second injection, and 10 nmol/kg for the remaining four injections. Semaglutide treatment did not reduce body weight in male or female mice (Figure 4A,B). Food intake per mouse in group-housed cages receiving the same treatment on injection day was also not reduced by semaglutide (Figure 4C,D). At the end of the treatment period, fasting blood glucose was not affected by semaglutide. Blood glucose levels were significantly lower in vehicle-treated females (*p* = 0.018) compared to vehicle-treated males (Figure 4E). Semaglutide significantly lowered fasting serum insulin levels in females in comparison to semaglutide-treated males (*p* = 0.009) and vehicle-treated females (*p* = 0.036, Figure 4F). Vehicle-treated females also had significantly lower serum insulin levels compared to vehicle-treated males (*p* < 0.0001, Figure 4F). Fasting serum leptin showed a trend towards lower levels in response to semaglutide (*p* = 0.068, Figure 4G). Semaglutide significantly reduced fasting serum GLP-1 in females (*p* = 0.043), but not males (Figure 4H). These data indicate that lean females may be more metabolically sensitive to IRA treatment than lean males.

**Figure 5:**
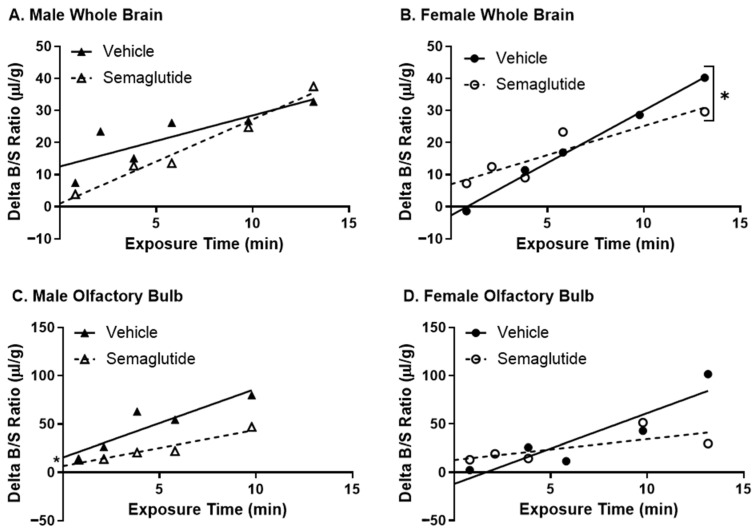
Effect of chronic semaglutide treatment on IV ^125^I-insulin BBB pharmacokinetics. Delta ^125^I-insulin BBB pharmacokinetics were measured for (**A**,**B**) whole brain (*n* = 5–6) and (**C**,**D**) olfactory bulb (*n* = 5) in male and female mice following two weeks of s.c. semaglutide (dose escalation 1–10 nmol/kg) treatment. * *p* < 0.05 between slopes (*K_i_*).

Following chronic semaglutide treatment, we investigated ^125^I-insulin BBB pharmacokinetics (Figure 5A,B, Table 4). Semaglutide reduced insulin BBB transport into the whole brain in females (*p* = 0.025), but not in males. Insulin transport rates into the olfactory bulb trended towards reduction in females (*p* = 0.063) (Figure 5D). Although there were no significant differences in insulin BBB transport rates for males, there was a significant reduction in the level of reversible binding for insulin in the olfactory bulb (*p* = 0.011) (Figure 5C).

**Figure 6:**
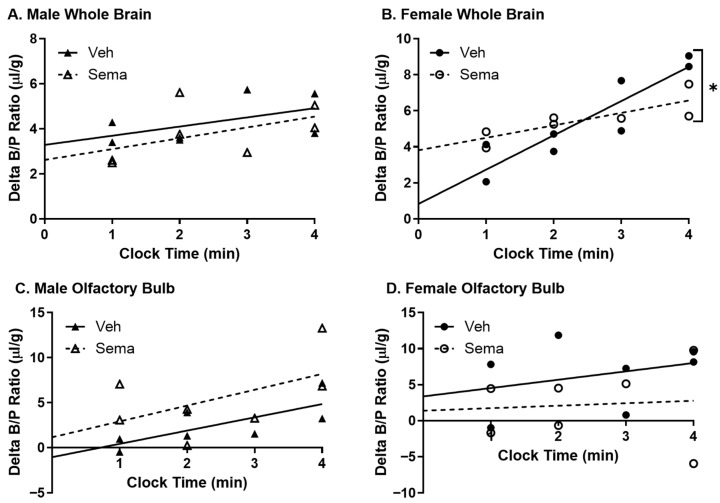
Effect of semaglutide treatment on in situ ^125^I-insulin BBB pharmacokinetics. Delta ^125^I-insulin pharmacokinetics were measured in the absence of blood (in situ) for (**A**,**B**) whole brain (*n* = 7–8) and (**C**,**D**) olfactory bulb (*n* = 7) in male and female mice following two weeks of s.c. semaglutide (dose escalation up to 10 nmol/kg) treatment. * *p* < 0.05 between slopes (*K_i_*). Veh: vehicle, Sema: semaglutide.

Insulin BBB transport is a saturable process, meaning that increasing levels of insulin in the circulation can reduce insulin BBB transport rates. We observed significant reductions in serum insulin levels, particularly in females, following chronic semaglutide treatment (Figure 4F). Therefore, we wanted to re-examine insulin BBB transport in the absence of circulatory factors such as insulin, which could act as inhibitors or allosteric regulators. Following chronic semaglutide treatment, we performed in situ perfusion studies, eliminating any effects of serum factors. Insulin BBB transport into the whole brain was still reduced in females following chronic semaglutide treatment (*p* = 0.016) and remained unaltered in males (Figure 6A,B; Table 5). The delta brain/perfusion data for the olfactory bulb was too variable to significantly calculate transport rates in both males and females, and so statistical analyses could not be performed (Figure 6C,D; Table 5). These data indicate that chronic IRA treatment is directly affecting the function of the BBB in females.

## 3. Discussion

Here, we expanded studies on the regulation of IRA transport across the BBB, as well as examined the effects of IRAs on the BBB transport of insulin. By administering the insulin receptor antagonist S961 into the brain’s lateral ventricle, we found evidence that acute changes in brain insulin signaling do not regulate IRA transport into the brain. In contrast, we identified that systemic insulin enhances IRA transport into the brain. Whereas acute IRA treatment had minimal effects on insulin BBB pharmacokinetics, chronic IRA treatment significantly reduced insulin BBB transport, but in females only. These findings expand our understanding of IRA BBB transport regulation and highlight a functional, sex-selective effect on the BBB by IRA treatment. This work is critical to understanding the mechanisms of action of IRAs in the brain.

The regulation and relevance of IRA entry into the brain is an active area of investigation. While it seems as though the first class of IRA therapies (e.g., exenatide and the closely related dulaglutide (BAF)) cross the BBB rapidly, the next generation with increased residence times in blood (e.g., liraglutide, semaglutide) has more difficulty navigating the BBB [17]. Expanding on transport regulation, most studies indicate that the GLP-1 receptor at the BBB is not involved in transport. First, IRA BBB transport is not saturable at physiological doses [16,19], which is a main feature of receptor-mediated transport. Instead, we identified that non-acylated IRAs, including dulaglutide (BAF), cross by virtue of their lipid solubility [17]. Second, brain-specific sequencing data sets, including those enriched for vascular cells, indicate that the GLP-1 receptor is not broadly expressed in brain endothelial cells, but rather may be more localized to vascular smooth muscle cells [20,21]. However, given that brain expression of the GLP-1 receptor is low, in an attempt to enrich the cell population of interest, one recent study used a unique approach to first isolate GLP-1 receptor-expressing cells from the hypothalamus followed by single-cell sequencing, which identified a cluster of endothelial cells [22]. It should be noted that this study relied on genetic expression of an endogenous fluorescent reporter, which can result in aberrant expression or artifacts. Stronger evidence exists for the role of the GLP-1 receptor in mediating transport across the tanycytic barrier between the median eminence and the hypothalamus [23]. Ultimately, the lack of GLP-1 receptors in brain endothelial cells would be consistent with recent reanalyzed data showing that lipid solubility is a major mechanism by which those IRAs that can cross the BBB do so [17]. However, there is evidence that some brain endothelial cells, such as those from the rat, do have GLP-1 receptors and a saturable component to BBB transport [24].

Seeking to examine the regional transport capabilities of IRAs, we identified that dulaglutide (BAF) transport rates varied regionally by nearly 2.5-fold. Using fluorescent or even newly developed PET radiotracers, prior studies indicate that regional appearance of the albumin-binding IRAs (including semaglutide, liraglutide and tirzepatide) is limited to circumventricular organ regions [23,25,26]. In our studies, hypothalamic delta B/S ratios were too variable to calculate an accurate transport rate. This could be due to the variability of transport into the various nuclei that comprise the hypothalamus and the role of IRAs or GLP-1 receptors within each nucleus. In comparison, prior studies have shown that insulin BBB transport is typically greatest for the olfactory bulb and pons as well, with regional rates varying by about 3.5-fold [27,28]. The faster transport rates into these regions could be driven by the functional effects of IRAs within each region. For example, the olfactory bulb is a metabolic regulator of energy homeostasis, modulating odor-driven food intake and metabolism [29,30]. The pons/medulla, or hindbrain, is involved in visceral feedback for suppressing food intake and reducing appetite [31,32]. Both regions exhibit high expression of GLP-1 and GIP receptors. The hypothalamus and hippocampus are next, with the midbrain and parietal cortex exhibiting the slowest rates of transport. Insulin BBB transport into the thalamus is typically undetected. With the development of PET-IRAs, we will be able to examine GLP-1 and insulin transport properties in humans, gaining greater mechanistic and translational insights.

To further examine the regulation of BBB permeation for IRAs, we explored two additional points of regulation: (1) the role of a negative feedback loop and (2) cross-regulation. First, as IRAs are being investigated for the treatment of AD and IRAs reverse dysregulated brain insulin signaling in AD, we wanted to examine whether dysregulated brain insulin signaling impacted the ability of IRAs to cross the BBB. We have previously identified a negative feedback regulation for insulin BBB transport [18]. In our current study, we found that acute inhibition of brain insulin receptors did not affect the BBB permeation of dulaglutide (BAF). We have not yet determined whether a negative feedback mechanism exists between GLP-1 receptor signaling and the rate of IRA BBB transport, making this an important area for future investigation. Second, we investigated whether insulin could regulate IRA BBB transport and vice versa. While quickly transported IRAs are regionally regulated by insulin, they do not regulate insulin BBB transport. However, we did find that dulaglutide (BAF) reduced insulin binding at the BBB in the whole brain of male mice. These data suggest that dulaglutide (BAF) treatment may rapidly affect the presence of insulin receptors at the cell surface of the BBB or impact insulin’s ability to interact with the BBB insulin receptor. Similar, but non-significant, results were found in the olfactory bulb.

While acute dulaglutide (BAF) did not affect insulin BBB transport rates, insulin did increase dulaglutide (BAF) BBB transport rates. Another study recently reported that insulin increased fluorescent exendin-4 appearance in the hypothalamus via a peripheral insulin receptor-dependent mechanism [33]. Regulation of dulaglutide (BAF) BBB transport by insulin indicates that therapies designed to mimic endogenous metabolic hormones can inadvertently impact BBB function, including the transport of other key metabolic hormones. Additional work is needed to identify not only the mechanisms for altering BBB function, but also the extent of these changes by investigating the BBB transport of other metabolic hormones such as leptin, ghrelin, or amylin. Although prior work has focused on the changes in exendin-4 transport into the hypothalamus [33], our work extends these findings to other brain regions and supports the idea that changes in energy needs alter IRA BBB transport. Whether these changes are due to receptor desensitization, transporter modulation, or endothelial signaling remains to be determined. There is evidence indicating that IRAs cross the BBB through adsorptive transcytosis [17]. Whether changes in cerebral blood flow can impact adsorptive transcytosis is unclear. Nevertheless, it is unlikely that cerebral blood flow is involved in increasing dulaglutide (BAF) BBB transport in our system, as intravenous insulin has previously been shown to not alter cerebral blood flow based on prior work [34]. These changes could have important translational impacts on human metabolism and neurocognitive function. Overall, these findings support independently regulated mechanisms of transport between insulin and IRAs.

Acute treatments allow us to better interpret chronic effects, as they are often the first initiator of what may compound over time with chronic treatment. To examine the chronic effects of IRAs on the BBB with a drug that is commonly used in the clinic, we investigated the effects of chronic semaglutide treatment on BBB function. In obese rodents, semaglutide takes effect within 24 h of treatment, reducing food intake and body weight [25,35]. Reductions in body weight typically plateau by 12 days in mice [25]. There is less literature available on the effects of semaglutide on body weight in lean, healthy subjects, as the majority of studies have focused on individuals with type 2 diabetes, obesity, or overweight. In the EVOKE trial, consisting of individuals with MCI ages 55–85 years and older, 104 weeks of semaglutide treatment resulted in a mean body weight loss of 5.8%, with the largest changes occurring in individuals with a BMI greater than 30. Two studies treating lean male mice for up to 5 weeks with 200 µg/kg daily semaglutide found no effect on body weight [36,37]. Our current findings are in line with these previous reports, indicating that male or female mice treated with up to ~50 µg/kg semaglutide three times per week had no effect on body weight or food intake. Importantly, our work extends these findings to female mice. Despite the lack of reductions in body weight, semaglutide reduces fasting serum insulin and GLP-1 levels, but only in females, independent of changes in fasting blood glucose. These metabolic measurements are critical to our interpretation given that these changes occur in the absence of changes in body weight or food intake.

Finally, we examined the chronic effects of semaglutide treatment on BBB transport function. Prior reports indicate that IRA treatment in humans enhances BBB transport function by measuring brain glucose uptake in both healthy controls and individuals with AD. Using FDG-PET, acute IV synthetic GLP-1 (7–36) (0.25 mg) increased blood-to-brain glucose transport in hyperglycemic, but not hypoglycemic, fasted healthy men [38]. In a small cohort consisting of similar numbers of male and female individuals with AD, daily liraglutide treatment up to 1.8 mg/day for 6 months maintained brain glucose uptake similar to that of healthy controls [39]. Whether these are due to functional effects at the BBB or simply changes in cerebral blood flow, which would alter flow-dependent glucose uptake, remains to be determined. We identified that chronic IRA treatment affected the function of the BBB by reducing insulin BBB transport in whole brain, but only in female mice at the tested dose. These reductions were not due to circulatory factors, as we observed the same reduction when blood was eliminated from our system by using the in situ brain perfusion method. Insulin BBB transport is affected by multiple circulating factors, including triglycerides and even insulin itself [40]. Chronic semaglutide clearly affected the circulating factors of some hormones measured in this study, decreasing insulin and GLP-1 levels in lean female mice. While a reduction in insulin would increase the transport of the insulin radiotracer due to a lack of competition, we saw the opposite, where insulin BBB transport was reduced. Our in situ studies supported this semaglutide reduction of insulin BBB transport, indicating that something other than a change in circulating factors is responsible. The reductions in insulin BBB transport following semaglutide treatment were contrary to what we hypothesized. While reductions in insulin BBB transport in disease states are often described as negative, the full picture remains to be elucidated. Insulin BBB transport is clearly finely tuned and is regulated by multiple pathways [41]. Determining the molecular mediators of insulin BBB transport will aid in understanding how this transport is impacted by various physiological states. IRAs are known to enhance brain insulin signaling [9,10,11]. We have shown that activation of insulin signaling downregulates insulin BBB transport [18]. Therefore, it is possible that signaling changes within the brain are driving the reductions in insulin BBB transport. However, this regulation was only detected in females, as we found no significant change in insulin BBB transport rates in males.

Recent literature suggests that females exhibit greater weight loss, reductions in diastolic blood pressure, and experience more gastrointestinal side effects than males during IRA treatment [42,43,44]. Additional studies have examined the sex-specific impact on co-morbid outcomes, including cardiovascular and renal outcomes, and found no differences between men and women taking IRAs [45]. Sex-specific cognitive differences have not been examined, and large-scale studies that have been performed were not powered to detect sex differences [46]. Why we detected IRA effects on BBB function in females only remains to be determined. Our perfusion data suggests that IRAs are altering molecular factors specifically at the BBB, but only in females. It is unclear whether males and females are truly dichotomous, whether females are simply more sensitive, or whether males will also respond with different dosing regimens. By extension, there does not seem to be a sex-specific effect of IRAs on the structure of the BBB, as evident from a meta-analysis on stroke risk [45].While there needs to be more in-depth investigation into the sex differences as to why IRAs affect the function of the BBB by reducing insulin BBB transport, it is clear these sex differences should be taken into consideration when investigating potential mechanisms of IRAs.

These studies highlight that IRAs can alter properties of the BBB, affecting hormone BBB transport, specifically for insulin, with females being more sensitive to these changes. Alternatively, insulin was also able to affect IRA BBB transport, highlighting reciprocal interaction and regulatory properties. The exact mechanisms governing these changes in the functional activities of the BBB remain to be determined.

## 4. Methods

### 4.1. Incretin Peptide Sources

Dulaglutide (BAF) (3315 Da, GC31520, GLPPBIO, Montclair, CA, USA) and semaglutide (4114 Da, 4091661, Bachem, Torrance, CA, USA) were commercially purchased.

### 4.2. Animals

All studies used two-month-old lean male and female CD-1 mice purchased from Charles River Laboratories (Seattle, WA, USA). The mice had ad libitum access to food and water and were kept on a 12/12 h light/dark schedule, with the light phase beginning at 6:00 AM daily. Mouse body weights were recorded at the beginning of each study, on each injection day, and on the day of the terminal study. For all terminal animal studies, the mice were anesthetized via intraperitoneal injection (IP) of 40% urethane to minimize discomfort and warmed on a heating pad while anesthetized to maintain their body temperature. All animal procedures were approved by the VA Puget Sound Institutional Animal Care and Use Committee (IACUC) and performed at an Association for Assessment and Accreditation of Laboratory Animal Care International (AAALAC) certified facility.

### 4.3. Semaglutide Treatment

For chronic IRA treatment, the lean mice were weighed and treated with subcutaneous (s.c.) semaglutide on a Monday, Wednesday, Friday schedule for two weeks, with treatment ending 48 h prior to the terminal study, for a total of 6 injections. A dose escalation of 1 nmol/kg, 3 nmol/kg, and then 10 nmol/kg of semaglutide (Bachem) or vehicle (0.05% Tween-20 tris-buffered saline, TBST) was used during the first three treatments, and 10 nmol/kg was given for the remaining treatment days. The s.c. dose and 2-week treatment duration were selected based on prior work in obese rodents showing a significant reduction in body weight that plateaued at 12 days [25]. Given that we saw no signs of gastrointestinal distress or changes in weight in the first cohort, subsequent cohorts started at a 3 nmol/kg dose and escalated to 10 nmol/kg. Lean mice were used due to the accumulating evidence about the beneficial effects of IRAs in multiple diseases co-morbid with obesity. As the number of studies in lean individuals with these diseases is limited, a greater understanding of the effects of IRAs independent of obesity is needed. Body weight and food intake were measured on each injection day. As the mice were group-housed, food intake data is reported as food intake/day/mouse. On the day of tissue collection or of the terminal radioactive study, the mice were fasted for 6 h (6:00 am–12:00 pm) before conscious fasting blood glucose was assessed with a handheld glucometer (Abbott, Abbott Park, IL, USA) using blood obtained by tail prick. Final body weight was measured on this day.

### 4.4. Radioactive Labeling

For all studies involving radioactive tracers, insulin, dulaglutide (BAF), and albumin were radioactively labeled to aid in the measurement of BBB transport pharmacokinetics, while minimizing the necessary concentration of insulin or dulaglutide (BAF) administered needed for detection. Ten μg of human insulin (Sigma-Aldrich, St. Louis, MO, USA) or dulaglutide (BAF) in 0.25 M chloride-free sodium phosphate buffer (PB, pH 7.5), was combined with one millicurie (mCi) of ^125^I (Perkin Elmer, Waltham, MA, USA). Ten μg of chloramine-T (Sigma-Aldrich) was added to start the radioactive labeling reaction. Sodium metabisulfite (Sigma-Aldrich) (100 µg in 10 µL) terminated the reaction after the 60-s reaction time. Technetium-99m (^99m^Tc, Radioisotope Life Sciences, Seattle, WA, USA) was used to label bovine serum albumin (BSA) (Sigma-Aldrich). This was done in a 20-min reaction containing 1 mCi of ^99m^Tc, 1 mg albumin, 0.5 mL deionized water, 120 µg stannous tartrate, and 20 µL 1 M HCl. ^125I^-Insulin, ^125I^-dulaglutide (BAF), and ^99m^Tc-albumin were purified via a Sephadex G-10 (Sigma-Aldrich) column. From this column, 100 µL aliquots were collected in 100 µL of 1% BSA/lactated Ringer’s (BSA/LR). To determine protein binding for each isotope, precipitation with 30% trichloroacetic acid (TCA) was performed. Quality-control steps were performed on these radioactively labeled substrates to preclude overestimation of free radioactivity.

### 4.5. BBB Pharmacokinetics

For intravenous terminal radioactive studies, mice were anesthetized with approximately 150 µL of IP urethane (40%) and full anesthetic effect was verified with a lack of response to toe pinch. The jugular vein and the opposite carotid artery were exposed. The jugular vein was intravenously injected with 200 µL containing 1 × 10^6^ CPM ^125I^-insulin or -dulaglutide (BAF) and 500K CPM ^99m^Tc-albumin in 1% BSA/LR. In some studies, injections included 1 µg dulaglutide (BAF) or 1 µg insulin (co-administration studies). ^99m^Tc-albumin was included to measure and correct for vascular space. Samples were collected at 0.5, 1, 1.5, 2, 3, and 4 min after the intrajugular injection. Equal numbers of males and females were used. The carotid artery contralateral to the jugular vein receiving the injection was cut for blood collection, with subsequent decapitation. The olfactory bulb was weighed and removed, the whole brain was removed and dissected into brain regions after the method of Glowinski and Iverson [47] in some studies, and the regions were weighed. Serum was collected via arterial blood centrifugation at 3200 RCF for 10 min and 50 µL were transferred to a glass tube for radioactive counting.

For terminal in situ radioactive perfusion, a perfusate was prepared containing 200 K CPM/mL ^125^I-insulin and 100K CPM/mL ^99m^Tc-albumin in Zlokovic buffer (7.19 g/L NaCl, 0.3 g/L KCl, 0.28 g/L CaCl_2_, 2.1 g/L NaHCO_3_,0.16 g/L KH_2_PO_4_,0.17 g/L anhydrous MgCl_2_,0.99 g/L D-glucose, and 1% BSA). In anesthetized mice, the thoracic cavity was opened and non-radioactive arterial blood was collected from the descending aorta and placed on ice for 30 min, after which it was centrifuged for serum collection and frozen at −80 °C for subsequent biochemical analysis. The descending aorta was clamped, the jugular veins were cut, and a 26-gauge butterfly needle was inserted into the left ventricle of the heart. The perfusate flow rate was set to 2 mL per min, and the mice were perfused for 1, 2, 3, or 4 min. After perfusion, each mouse was decapitated for dissection of the olfactory bulb and whole brain. The samples were weighed and counted with background correction for 3 min (^125^I/^99m^Tc). Three perfusate checks (20 µL) were taken at the beginning, middle, and end of the study for both ^125^I and ^99m^Tc.

For all radioactive studies, samples were counted on background-counted racks for 3 min (^125^I/^99m^Tc). Three injection checks (20 µL) were taken throughout the entire study for dual ^125^I and ^99m^Tc counting. Each mouse used provided one serum sample and its corresponding time-matched brain sample.

Exposure time and unidirectional influx into the brain were calculated as previously described [48]. The numerical difference between the ^125^I-peptide brain/serum (B/S) or ^125^I-peptide brain/perfusate (B/*P*) (µL/g) ratio and that of ^99m^Tc-albumin B/S or B/*P* ratio provides a delta ^125^I-peptide B/S or B/*P* ratio corrected for vascular space. *K_i_* (µL/g-min) is the steady-state rate at which the peptide enters the brain from the serum. *V_i_* (µL/g) is the amount of peptide that is reversibly bound to the BBB.

### 4.6. Serum Hormone Assay

The serum samples were thawed, and serum insulin, leptin, and GLP-1 levels were assessed using the Bio-Plex Pro™ mouse diabetes immunoassay as per the diabetes plex manual. The standard diluent option was used for 4-fold diluted serum samples, producing an 8-point standard curve. Plates were read on a Bio-Plex 200 (Bio-Rad, Hercules, CA, USA). Out-of-range samples were not included in the final analysis.

### 4.7. Statistical Analysis

All statistical analyses were performed with Prism 10.0 (GraphPad Software Inc., San Diego, CA, USA). In all MTRA pharmacokinetic studies, the linear regression lines’ slope (*K_i_*) as well as their corresponding correlation coefficients (*r*) and y-intercepts (*V_i_*) were compared for statistical significance with Prism’s version of an analysis of covariance (ANCOVA). Means are reported with standard errors. Outliers were removed using the GROUT method. Two-way ANOVAs were used when comparing multiple variables. Statistical significance was defined as *p* < 0.05.

## 5. Conclusions

We have expanded our understanding of IRA transport into the brain, as well as the effects of IRAs on the BBB itself. IRA BBB transport is not impacted by dysregulated brain insulin signaling. However, it is regulated by metabolic hormones like insulin. Chronic IRA treatment reduces insulin BBB transport in females, but not in males. The mechanisms driving these changes at the BBB and the downstream implications for this require further investigation. Clearly, IRAs and insulin have interacting roles at the BBB and affect each other’s transport characteristics.

## Figures and Tables

**Table 1:** Dulaglutide (BAF) BBB pharmacokinetics following acute brain insulin receptor inhibition.

Tissue	Treatment	*K_i_* (uL/g-min)	*r*	*n*	*p K_i_*	*p*	*V_i_* (uL/g)	*p V_i_*
Whole Brain	Vehicle	1.24 ± 0.27	0.87	9	0.002	0.98	1.21 ± 0.83	0.17
	S961	1.19 ± 0.16	0.92	11	<0.0001		0.53 ± 0.56	
Olfactory Bulb	Vehicle	0.97 ± 0.49 (ns)	0.66	7	0.11	0.16	6.12 ± 1.62	0.52
	S961	1.88 ± 0.41	0.82	12	0.001		1.99 ± 0.41	

ns: non-significant linear regression; *r* is the correlation coefficient for the regression lines between time and brain region/serum ratios shown in Figure 1; *p K_i_* refers to the statistical comparison of the regression lines and *p* to the statistical comparison of *K_i_* or *V_i_* for Vehicle vs. S961.

**Table 2:** Dulaglutide (BAF) BBB pharmacokinetics with co-administered IV insulin.

Tissue	Treatment	*K_i_* (uL/g-min)	*r*	*n*	*p K_i_*	*p*	*V_i_* (uL/g)	*p V_i_*
WB	Vehicle	0.80 ± 0.22	0.77	11	0.0055	**0.029**	−1.41 ± 0.85	-
	Insulin	1.59 ± 0.25	0.89	12	<0.0001		−2.80 ± 0.95	
OB	Vehicle	1.38 ± 0.31	0.91	6	0.011	**0.001**	−0.59 ± 1.32	-
	Insulin	2.77 ± 0.18	0.98	12	<0.0001		−4.29 ± 0.66	
FCtx	Vehicle	1.38 ± 0.15	0.97	11	<0.0001	0.81	−1.80 ± 0.54	0.69
	Insulin	1.45 ± 0.19	0.93	12	<0.0001		−2.20 ± 0.70	
Str	Vehicle	0.76 ± 0.21	0.85	7	0.0149	0.99	−0.73 ± 0.75	0.54
	Insulin	0.75 ± 0.21	0.77	12	0.0053		0.75 ± 0.78	
Hypo	Vehicle	−0.28 ± 1.64 (ns)	0.05	12	0.8656	na	7.44 ± 6.15	na
	Insulin	1.15 ± 1.42 (ns)	0.25	12	0.4382		1.15 ± 5.34	
Hippo	Vehicle	0.98 ± 0.43	0.59	12	0.0441	0.56	−4.32 ± 1.60	**0.02**
	Insulin	1.29 ± 0.32	0.79	12	0.0023		2.53 ± 1.19	
Thal	Vehicle	0.74 ± 0.27	0.66	12	0.0207	0.42	−2.66 ± 1.01	0.13
	Insulin	1.06 ± 0.27	0.77	12	0.0031		−2.36 ± 1.01	
PCtx	Vehicle	0.57 ± 0.21	0.64	12	0.0249	**0.026**	−1.08 ± 0.80	-
	Insulin	1.23 ± 0.17	0.91	12	<0.0001		−2.50 ± 0.65	
OCtx	Vehicle	0.96 ± 0.33	0.70	11	0.017	0.062	−2.80 ± 1.29	**0.03**
	Insulin	2.36 ± 0.60	0.78	12	0.0029		−3.52 ± 2.26	
CB	Vehicle	1.35 ± 0.29	0.83	12	0.0008	0.59	−3.12 ± 1.08	0.34
	Insulin	1.55 ± 0.22	0.91	12	<0.0001		−3.02 ± 0.81	
Mid	Vehicle	0.88 ± 0.33	0.65	12	0.0233	0.42	−2.26 ± 1.24	0.32
	Insulin	1.23 ± 0.27	0.82	12	0.001		−2.43 ± 1.00	
Pons	Vehicle	1.21 ± 0.42	0.67	12	0.0163	**0.019**	−3.49 ± 1.57	-
	Insulin	2.87 ± 0.50	0.88	12	0.0002		−5.52 ± 1.88	

ns: non-significant linear regression; WB: whole brain, OB: olfactory bulb, FCtx: frontal cortex, Str: striatum, Hypo: hypothalamus, Hippo: hippocampus, Thal: thalamus, PCtx: parietal cortex, OCtx: occipital cortex, CB: cerebellum, Mid: midbrain, Pons: pons/medulla. *r* is the correlation coefficient for the regression lines between time and brain region/serum ratios shown in Figure 2; *p K_i_* refers to the statistical comparison of the regression lines and *p* to the statistical comparison of *K_i_* or *V_i_* for Vehicle vs. Insulin. Values in bold represent statistically significant comparisons between groups.

**Table 3:** Insulin BBB pharmacokinetics with co-administered IV dulaglutide (BAF).

Tissue	Treatment	*K_i_* (uL/g-min)	*r*	*n*	*p K_i_*	*p*	*V_i_* (uL/g)	*p V_i_*
Whole Brain	Vehicle	1.01 ± 0.32	0.71	12	0.0099	0.49	4.95 ± 1.74	**0.02**
	Dula	0.74 ± 0.22	0.23	12	0.0064		2.20 ± 2.12	
Olfactory Bulb	Vehicle	2.16 ± 0.62	0.76	11	0.0069	0.99	3.71 ± 3.51	0.17
	Dula	1.98 ± 0.71	0.70	10	0.0238		−2.18 ± 5.94	

*r* is the correlation coefficient for the regression lines between time and brain region/serum ratios shown in Figure 3; *p K_i_* refers to the statistical comparison of the regression lines and *p* to the statistical comparison of *K_i_* or *V_i_* for Vehicle vs. Dula. Values in bold represent statistically significant comparisons between groups. Dula: dulaglutide (BAF).

**Table 4:** IV insulin BBB pharmacokinetics following chronic semaglutide treatment.

Tissue	Sex	Treatment	*K_i_* (uL/g-min)	*r*	*n*	*p K_i_*	*p*	*V_i_* (uL/g)	*p V_i_*
Whole Brain	Male	Vehicle	1.59 ± 0.54	0.83	6	0.042	0.15	12.55 ± 3.96	0.14
		Sema	2.63 ± 0.25	0.99	5	0.002		0.98 ± 2.01	
	Female	Vehicle	3.27 ± 0.132	1.00	5	0.0001	**0.03**	−2.68 ± 1.05	-
		Sema	1.82 ± 0.47	0.91	5	0.031		7.01 ± 3.18	
Olfactory Bulb	Male	Vehicle	7.09 ± 1.88	0.91	5	0.033	0.14	15.59 ± 10.28	**0.01**
		Sema	3.73 ± 0.67	0.96	5	0.011		6.67 ± 3.65	
	Female	Vehicle	7.32 ± 1.97	0.91	5	0.034	-	−11.96 ± 15.68	-
		Sema	2.16 ± 1.20 (ns)	0.72	5	0.171		12.82 ± 9.14	

ns: non-significant linear regression; *r* is the correlation coefficient for the regression lines between time and brain region/serum ratios shown in Figure 5; *p K_i_* refers to the statistical comparison of the regression lines and *p* to the statistical comparison of *K_i_* or *V_i_* for Vehicle vs. Sema. Values in bold represent statistically significant comparisons between groups. Sema: semaglutide.

**Table 5:** In situ insulin BBB pharmacokinetics following chronic semaglutide treatment.

Tissue	Sex	Treatment	*K_i_* (uL/g-min)	*r*	*n*	*p K_i_*	*p*	*V_i_* (uL/g)	*p V_i_*
Whole Brain	Male	Vehicle	0.41 ± 0.29 (ns)	0.53	7	0.225	0.88	3.29 ± 0.79	0.38
		Sema	0.48 ± 0.37 (ns)	0.51	7	0.245		2.62 ± 0.99	
	Female	Vehicle	1.90 ± 0.35	0.91	8	0.002	**0.02**	0.83 ± 0.97	-
		Sema	0.69 ± 0.22	0.82	7	0.024		3.81 ± 0.58	
Olfactory Bulb	Male	Vehicle	1.47 ± 0.59 (ns)	0.74	7	0.055	-	1.05 ± 1.60	-
		Sema	1.76 ± 1.24 (ns)	0.54	7	0.216		1.16 ± 3.35	
	Female	Vehicle	1.16 ± 1.56 (ns)	0.31	7	0.491	-	3.38 ± 4.42	-
		Sema	0.34 ± 1.84 (ns)	0.08	7	0.859		1.40 ± 4.97	

ns: non-significant linear regression; *r* is the correlation coefficient for the regression lines between time and brain region/perfusate ratios shown in Figure 6; *p* K_i_ refers to the statistical comparison of the regression lines and *p* to the statistical comparison of *K_i_* or *V_i_* for Vehicle vs. Sema. Values in bold represent statistically significant comparisons between groups. Sema: semaglutide.

## Data Availability

The datasets supporting the conclusions of this article are included within the article. Further inquiries can be directed to the corresponding author.
